# Biological Systems Depend on Communication Over Distances: A Review of the Fundamental Mechanisms, Associated challenges, and the Potential Effect of the Constrained Disorder Principle

**DOI:** 10.1007/s10441-026-09515-w

**Published:** 2026-02-01

**Authors:** Yaron Ilan

**Affiliations:** 1 Department of MedicineHadassah Medical Center, and Faculty of Medicine, Hebrew University, Jerusalem, Israel; 2 Department of Medicine, Hadassah Medical Center, POB 1200, IL91120 Jerusalem, Israel

**Keywords:** Distance biology, Constrained disorder principle, Consciousness, Water, Immunology, Electromagnetic fields, Morphic resonance, Quantum field effects

## Abstract

Biological systems depend on communication over distances, ranging from molecular gradients to systemic neuroendocrine and neuroimmune circuits. While many distance effects in biology are explained by well-established mechanisms such as diffusion, paracrine signaling, neural conduction, and extracellular vesicle trafficking, there are also claims of long-distance influences that may be mediated by consciousness, electromagnetic fields, or hypothesized morphic fields. This review synthesizes controlled laboratory evidence, evaluates speculative mechanisms, including quantum field effects and morphic resonance, and compares them with well-replicated findings in immunology and bioelectromagnetics. The Constrained Disorder Principle (CDP) is a novel theoretical framework that posits variability within constraints as essential for biological function and may underlie some of these effects. The paper discusses the debate over methodological rigor and replicability in research on nonlocal biological effects. While evidence supports the importance of distance in biological communication through known carriers, claims regarding consciousness and morphic resonance remain unverified, despite challenges to their validity. Future research must strike a balance between openness and rigorous experimental standards.

## Introduction

Distance is a fundamental aspect of biological communication. Organisms rely on mechanisms that propagate effects beyond the immediate vicinity of a cell or tissue, from molecular diffusion to hormonal signaling(Su et al. 2024; Ullo and Case 2023; McMillen et al. 2021). For example, morphogen gradients specify positional information during development, chemokines direct leukocyte migration in immunology, and the nervous system and endocrine hormones relay signals across entire organ systems(Powell et al. 2017; Huizar et al. 2020; Ulrich-Lai and Herman 2009). Such distance effects are well established in the experimental literature (Alisafaei et al. 2021).

**Mechanistic explanations** in biology typically involve identifying the entities (molecules, cells, organs) and activities (binding, signaling, transport) that produce a phenomenon, along with their spatial and temporal organization. Such explanations answer “how” questions by describing the causal pathways that link components across different levels of biological organization. However, there has also been a long-standing interest in possible nonlocal effects; for instance, some claim that consciousness can affect water crystallization(Emoto 2004), influence optical interference patterns(Radin et al. 2012), or impact biological systems more generally(Earl 2014; Tononi and Koch 2015). Other hypotheses, such as Rupert Sheldrake’s morphic resonance(Gomez-Marin 2021) and quantum field-based models(Hameroff and Penrose 2014; Penrose 2001), seek to explain these long-distance, non-mechanistic effects. These proposals challenge conventional mechanistic frameworks by suggesting information transfer or correlations that operate outside known physical carriers or contact-dependent processes.

The study of distance effects in biology encompasses a spectrum from rigorously established phenomena to highly disputed claims. Understanding this continuum requires careful examination of experimental methodology, replication standards, and the theoretical frameworks employed to explain observed effects(Laraway et al. 2019; Nosek et al. 2015).

This review focuses specifically on **inter-organ and inter-cellular distance effects** where communication occurs over millimeter-to-meter scales, with particular emphasis on immunological and electromagnetic phenomena. These systems were selected because: (1) they represent well-characterized examples of legitimate biological distance effects, (2) they provide a framework for evaluating more controversial claims, and (3) recent provocative findings suggest novel nonlocal correlations in immune system components that may require new theoretical frameworks to explain.

A central organizing principle of this review is the **Constrained Disorder Principle (CDP)**, a theoretical framework that proposes all biological systems are characterized by controlled variability within dynamic boundaries. The CDP suggests that biological function depends on maintaining appropriate levels of disorder constrained within specific limits. This framework may help explain how local perturbations propagate across biological systems. The CDP offers a potentially unifying perspective on distance effects ranging from molecular allostery to ecosystem dynamics, and provides the theoretical basis for interpreting the novel experimental findings on nonlocal immune correlations.

This paper reviews several controlled laboratory findings, proposed mechanisms, and debates surrounding replication, with a focus on immunological and electromagnetic distance effects, as well as more controversial claims related to consciousness-based influences. The goal of this review is to assess current knowledge while upholding scientific rigor when evaluating extraordinary claims. **Explicit criteria for scientific demarcation** will be applied throughout, including: (1) reproducibility by independent laboratories, (2) appropriate statistical controls and pre-registration, (3) plausible physical mechanisms consistent with established principles, (4) effect sizes that exceed measurement error, and (5) resistance to more straightforward alternative explanations such as experimental artifacts or investigator bias.

## Distance Effects in Biology: Mechanistic Foundations

### Molecular Diffusion and Concentration Gradients

The most fundamental distance effect in biology is molecular diffusion, which is governed by Fick’s laws and thermodynamic principles(Kinsey et al. 2011). Mathematical modeling of diffusion processes indicates that the effective signaling distance is proportional to the square root of the diffusion coefficient-to-degradation rate ratio (Berezhkovskii et al. 2010). For typical signaling molecules, this restricts paracrine communication to distances of about 25 cell diameters, or approximately 500 micrometers, in most tissues (Handly et al. 2015; Roy and Kornberg 2015). Beyond this range, alternative mechanisms such as active transport, convective flow, or relay systems are necessary for effective communication(Tiayo et al. 2025; Kornberg and Roy 2014; Kornberg 2014). Diffusion-mediated gradients are fundamental to paracrine, endocrine, and synaptic signaling, though they operate at characteristic spatial and temporal scales in each context (Nguyen et al. 2015).

### Paracrine Signaling Networks

Paracrine signaling involves cells communicating with neighbors by secreting soluble factors (Francis and Palsson 1997), with effective distances limited to around 25 cell diameters (Francis and Palsson 1997). Recent research shows that while paracrine signaling can reduce variability in cellular responses, variability commonly persists (Handly et al. 2015; Walker et al. 1999), highlighting the complex balance between coordination and independence in cellular networks (Jordan et al. 2000). Paracrine signals typically elicit rapid, short-lived responses, with rapid degradation ensuring signal fidelity (Handly et al. 2015; Roy and Kornberg 2015).

### Endocrine, Neural, and Extracellular Vesicle Systems - Condensed

Endocrine signaling extends communication across entire organ systems via the circulation (Kress and Mennerick 2009; Zhu et al. 2021), with hormones maintaining specificity through receptor-mediated recognition and operating on timescales of hours to days (Walker et al. 1999; Sellegounder et al. 2023). Neural systems achieve near-instantaneous communication at speeds up to 120 m/s(Kress and Mennerick 2009), integrating multiple distance scales from synaptic clefts to meter-scale projections(Zhu et al. 2021; Munn et al. 2024). Extracellular vesicles provide a “packaging” mechanism for transporting complex molecular cargo over considerable distances(Tetta et al. 2013; Bella and Taverna 2024; Yáñez-Mó et al. 2015; Kumar et al. 2024).

## Consciousness and Water: Emoto and Radin Studies

### Historical Context and Claims

Masaru Emoto’s claims regarding the effects of consciousness on water crystallization have attracted attention(Emoto 2004; Radin et al. 2008a, b), proposing that intentions influence ice crystal formation—positive intentions producing aesthetically pleasing structures. These claims have been criticized as “exhaustive but wildly unscientific research“(Shang et al. 2005; Fisher and Homeopathy 2006).

### Methodological Concerns and Criticisms

**Applying scientific demarcation criteria**: Emoto’s work fails multiple standards of scientific rigor: **Reproducibility**—independent replications have yielded negative results(Matos et al. 2017; Radin et al. 1969). **Controls**—lack of blinding and inadequate sample sizes(Salerno 2014; Pitkanen 2018); (3) **Mechanism**—no plausible physical pathway(Pepperell 2018); (4) **Alternative explanations**—biased selection or selective reporting better explain observations.

Despite these flaws, Radin and colleagues conducted more rigorous double-masked tests with ~ 2,000 participants, finding treated crystals more aesthetically appealing (*p* = 0.003) (Radin et al. 2006). A follow-up triple-blind study showed similar results(Radin et al. 2008a, b). However, statistical significance alone does not establish causation. The modest effect size and lack of independent replication by other research groups mean these findings remain provisional and require substantial further verification before acceptance.

### Physical Mechanisms and Theoretical Challenges

Crystallization is governed by well-established thermodynamic and kinetic principles(Rupp 2015). The hypothesis that consciousness could influence this process would require energy transfer or field effects that are not demonstrated by conventional measurements (Pepperell 2018). The absence of a plausible mechanism, combined with failure of independent replication, leads to the provisional conclusion that reported effects likely represent experimental artifacts—though this conclusion remains open to revision with compelling new evidence meeting the criteria outlined above.

## Electromagnetic Fields as Mediators of Biological Distance Effects

### Endogenous Bioelectricity

All living organisms generate electromagnetic fields through action potentials, metabolism, and tissue currents (Nuccitelli 2003; Eskandani and Zibaii 2024), playing crucial roles in development, wound healing, and neural function (Sun et al. 2022; McCaig et al. 2005). Developmental bioelectricity involves coordinated voltage patterns that guide morphogenesis (Rajalekshmi and Agrawal 2024; Bartolomeo et al. 2022; Bassett et al. 1982), demonstrating that electromagnetic effects in biology are established mechanisms (Cifra et al. 2021; Funk et al. 2009; Nuccitelli 2003).

### ELF Fields, PEMF Therapy, Radiofrequency, and Magnetoreception

Extremely low-frequency fields have revealed both therapeutic applications and potential hazards(Eskandani and Zibaii 2024; Panagopoulos et al. 2025), though reproducibility remains debated(Kostoff and Lau 2013). Pulsed electromagnetic field therapy has demonstrated clinical efficacy in bone healing and pain management(Rajalekshmi and Agrawal 2024; Bartolomeo et al. 2022; Bassett et al. 1982; Mayer et al. 2024; Ross et al. 2019). Radiofrequency interactions exhibit well-understood thermal effects, while non-thermal effects remain controversial (Liu et al. 2024; Kim et al. 2019; Verschaeve et al. 2010). Biological magnetoreception in birds and turtles exemplifies an evolved sensitivity to weak electromagnetic fields (Panagopoulos et al. 2024; Kishkinev and Chernetsov 2015).

## Proposed Mechanisms: Quantum Field Models and Morphic Resonance

### Quantum Field Theory in Biology

Quantum effects are observed in specific processes, such as photosynthesis and enzyme catalysis (Maffei 2025). However, extending quantum principles to macroscopic biological organization faces significant challenges. Quantum coherence requires controlled conditions to prevent thermal decoherence (Valone and PRACTICAL CONVERSION OF ZERO-POINT ENERGY: 2003; Singh and Magnetoencephalography 2014), thereby fundamentally constraining its potential roles in biological communication (Matarèse et al. 2023).

### Morphic Resonance Hypothesis

Sheldrake’s morphic resonance proposes that biological systems influence similar systems across space and time through “morphic fields“(Gomez-Marin 2021; Sheldrake 1992; Azevedo et al. 2023). **Applying demarcation criteria**: This hypothesis makes testable predictions, but controlled experiments have generally failed to demonstrate proposed effects. The absence of supporting evidence and the lack of a plausible physical mechanism have led to widespread scientific skepticism (Shinnick et al. 2018; Myakishev-Rempel 2025). However, following Popper’s criterion of falsifiability, the hypothesis remains scientific precisely because it makes testable predictions—it is falsified by current evidence.

### Zero-Point Field and Coherent Field Theories

Zero-point field theories suggest that quantum vacuum fluctuations may mediate the transfer of biological information (Daneshpour and Youk 2019; Megha et al. 2021), but calculations show that these interactions are far too weak at physiological temperatures (Pophof et al. 2023; Milonni 1994; Škop et al. 2020; Weinberg 1989; Valone and PRACTICAL CONVERSION OF ZERO-POINT ENERGY: 2003). Coherent field models of consciousness propose that neural electromagnetic fields extend beyond the brain (Shabat et al. 2021; Ilan 2025a, b, c, d, e), but these fields decrease rapidly with distance, making external effects implausible (Singh and Magnetoencephalography 2014; Hosseini 2021; Næss et al. 2021).

## Distance Effects in Immunology

### Chemokine Gradients, Systemic Inflammation, and Neuroimmune Communication

The immune system relies on distance-dependent communication(Uhl and Gérard 2020; Poon and Farber 2020). Chemokine gradients guide immune cell migration at millimeter scales (Ilan 2022a, b, c, d, 2023a, b, c, d), while systemic inflammatory responses coordinate organism-wide effects through cytokine circulation (Navarro Quiroz et al. 2025; Daneshpour and Youk 2019; Megha et al. 2021; Evans et al. 2015; Valparaiso et al. 2014). Neuroimmune pathways, particularly the vagus nerve, provide rapid neural control of peripheral inflammation(Wang et al. 2025).

### Inter-Organ Correlations and System-Wide Communication

Recent research has uncovered new insights into inter-organ correlations, expanding the understanding of the effects of biological distance (Shabat et al. 2021, 2025; Shabat and Ilan 2021). Studies show that altering a single immune system element inevitably affects associated components, suggesting tightly coupled networks in which localized changes propagate to distant sites(Shabat et al. 2021, 2025; Shabat and Ilan 2021).

#### Novel Experimental Findings on Nonlocal Biological Interactions

The following studies represent the central empirical motivation for this review and the development of the CDP framework.

**Study 1 - Distant correlations in immune cells**: Using a murine model, researchers demonstrated that fasting and splenectomy induced measurable changes in immune cell populations located 10–20 cm away from the treated animals (Shabat and Ilan 2021). Immune cells were placed in tubes positioned above cages, eliminating conventional mediator transfer. The findings revealed: (1) trigger specificity—only certain biological states produced distant effects, and (2) cellular selectivity—specific immune cell types were responsive(Shabat and Ilan 2021). While significant effects were observed, future replications should specify exact p-values and effect sizes to enable meta-analysis.

#### Study 2 - Splenocyte Responses Without Contact

Splenectomy and fasting served as triggers, with splenocytes from operated mice placed in sealed tubes within cages(Ilan 2025a, b, c, d, e). Results showed in T cell subpopulations and cytokine secretion. Multiple pairwise comparisons revealed differences that approached but did not consistently meet conventional significance thresholds (*p* < 0.05). While such findings are typically not emphasized in original research papers due to insufficient statistical power, they are included here to provide complete transparency about the evidence base. Independent replication with larger sample sizes is needed to determine whether these represent genuine biological effects or type I errors. These borderline findings should be interpreted cautiously and are presented to stimulate further rigorous investigation rather than as definitive evidence.

#### Study 3 - Liver Inflammation Model

Mice injected with Concanavalin A (ConA) were housed with tubes containing livers from naïve mice, showing a significant reduction in alanine aminotransferase (ALT) serum levels. Conversely, housing with ConA-injected livers elevated ALT levels. Similar patterns were observed with splenocytes(Shabat et al. 2025). These findings suggest correlations between immune components using out-of-body organs without direct contact.

**Biological significance and open questions**: Currently, the functional role of these nonlocal interactions under normal physiological conditions remains unclear. Key questions include: (1) Under what natural conditions might such correlations occur? (2) What evolutionary advantage, if any, might they provide? (3) What specific features of biological systems enable these correlations? (4) Under what conditions can they be expected to occur versus not occur? These questions remain largely unanswered but provide direction for future research.

### Immune Memory and Long-Term Adaptation

Immunological memory involves temporal rather than spatial distance effects(Derksen et al. 2023). Memory cells distributed throughout the body retain the ability to mount rapid responses to previously encountered antigens, allowing the immune system to benefit from past experiences (Caserta et al. 2021). Maintaining immune memory requires complex regulatory networks that balance rapid responses against inappropriate activation (Hodgkinson et al. 2023; Schnaack and Nourmohammad 2021; Wang et al. 2020).

**Fig. 1:**
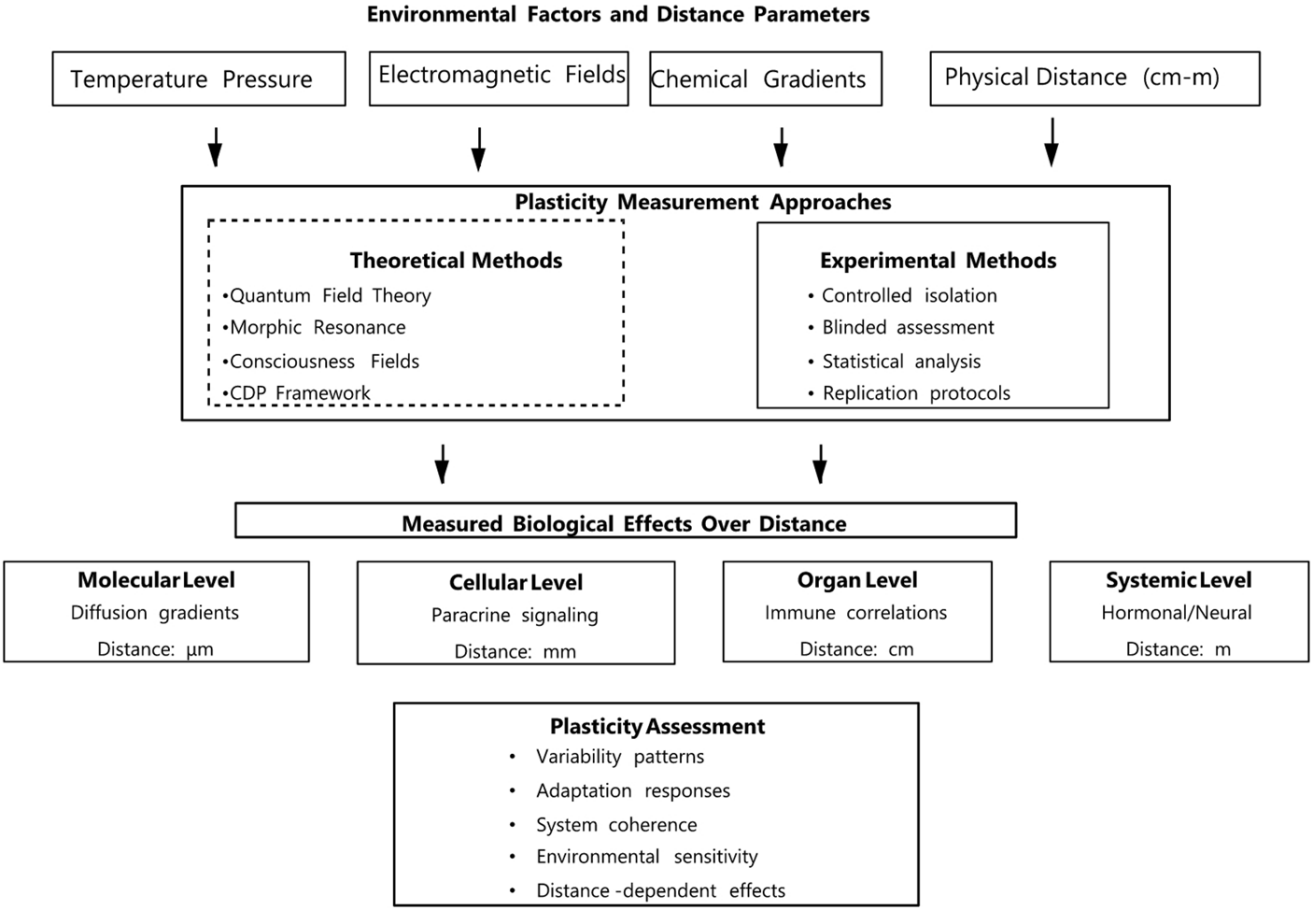
Assessing plasticity measures and their environmental relevance in remote influence effects: Theoretical and experimental methodologies for understanding distant influence

Figure 1 schematically illustrates various measures of plasticity and their environmental significance in evaluating the effects of distant influences, and presents several theoretical and experimental approaches to understanding remote impacts.

## The Constrained Disorder Principle May Explain the Effects of Distance

### Theoretical Framework of the CDP

The Constrained Disorder Principle (CDP) represents a novel theoretical framework for understanding biological complexity(Ilan 2022a, b, c, d).

**Core propositions**:

1. All biological systems are characterized by inherent variability constrained within dynamic boundaries.
2. This variability is not noise to be eliminated but essential for proper function.
3. Living systems differ from non-living systems in exhibiting higher variability within broader (but still constrained) boundaries.
4. System dysfunction arises from either insufficient variability or variability exceeding appropriate constraints.

The CDP draws on non-equilibrium thermodynamics and complex systems theory (Forkosh et al. 2020) and proposes that biological organization emerges from the strategic management of disorder within constraints (Ilan 2023a, b, c, d).

#### Boundaries and Constraints

The concept of “constraints” in CDP refers to dynamic limits on variability patterns rather than rigid mechanical constraints. Unlike fixed boundaries in closed physical systems, biological constraints are themselves adaptive, adjusting in response to internal and external pressures. It distinguishes CDP constraints from mechanical constraints in traditional mechanistic explanations — CDP constraints define permissible ranges of variability rather than deterministic causal pathways (Ilan 2023a, b, c, d, 2024a, b, c, d).

#### Systems and Boundaries

A key question is how to define system boundaries in the CDP framework. In statistical physics, boundaries are often arbitrary partitions for analytical convenience. In CDP, biological boundaries are defined functionally—by patterns of constraint and variability that maintain coherent function. This functional definition allows CDP to apply across scales from molecular to ecosystem levels.

The CDP defines all systems by their inherent variability(Ilan 2022a, b, c, d), proposing that variability is necessary for proper function and is dynamically adjusted in response to environmental and internal perturbations (here “pressures” refers to any forces—external or internal—that challenge system stability. It provides a unifying framework for understanding biological complexity and adaptability(Ilan 2024a, b, c, d, 2025a, b, c, d, e; Adar et al. 2025).

### Applications To Biological Systems

The CDP has been applied to various biological phenomena, from cellular to organ function, supporting the concept that disorder within dynamic boundaries constitutes biological order(Shabat et al. 2021; Ilan 2019a, b, c, d, e, 2020a, b, c, d, 2022a, b, c, d, 2023a, b, c, d; El-Haj et al. 2019; Ilan and Microtubules 2019; Ilan-Ber and Ilan 2019; Forkosh et al. 2020; Rotnemer-Golinkin and Ilan 2022). According to CDP, living organisms exhibit relatively high variability and dynamic borders, while non-living systems exhibit low variability and narrow borders. It provides a quantitative framework for distinguishing living from non-living systems. Variability characterizes the functionality of organs, the immune system, electromagnetic function, and water structure(Ilan 2023a, b, c, d).

### The CDP May Suggest that long-distance Effects in Biology

#### How CDP Explains

The CDP framework offers a distinct explanation of distance effects that differs from traditional mechanistic accounts. Rather than identifying discrete causal chains of molecular interactions, CDP explains through principles of **constraint propagation** and **variability correlation**.

When a local perturbation occurs in a CDP-governed system, it need not involve direct physical contact or mediator transfer to affect distant components. Instead, the perturbation alters the pattern of constrained variability. This modified pattern can propagate through the system via: (1) cascade effects utilizing inherent system fluctuations, (2) changes in constraint boundaries that affect distant subsystems coupled through the overall constraint network, and (3) correlation structures that link variability patterns across spatial scales.

This explanation is **testable** through specific predictions: (1) Distance effects should show trigger specificity—only perturbations that alter constraint patterns should produce correlations; (2) Effects should show cellular/tissue selectivity—only components within the same constraint network should respond; (3) Effect magnitude should depend on the degree of constraint coupling rather than physical proximity; (4) Blocking information flow should have less effect than disrupting constraint relationships.

#### Explanatory vs. Descriptive

The CDP aims to provide a genuine explanation rather than merely an accurate description. It proposes that distance correlations emerge *because* biological systems maintain function through constrained variability, rendering them a necessary consequence of living organization rather than an add-on mechanism. It differs from purely descriptive accounts that would document correlations without explaining why they exist.

The CDP proposes that living systems maintain functional organization by organizing disorder within constraints, leading to pattern emergence across extensive spatial and temporal scales (Ilan 2023a, b, c, d). According to CDP, system variability itself carries information—the pattern of fluctuations within constraints encodes functional state information that can be transmitted over distances (Ilan 2025a, b, c, d, e).

#### CDP and Information Propagation

The use of “information” in CDP contexts requires clarification. CDP uses information in the thermodynamic sense—as the degree of uncertainty or entropy within constrained boundaries. When distant biological systems show correlated variability patterns, they share informational structure even without direct physical communication channels. It aligns with concepts in non-equilibrium thermodynamics, in which systems can develop long-range correlations through constraint networks rather than through direct exchange of energy or matter(Zhang and Ouyang 2021).

#### Non-equilibrium Thermodynamics and Distance Correlations

How do non-equilibrium principles help understand “information propagation”? In systems far from equilibrium, local fluctuations can induce long-range correlations through the constraints that maintain the non-equilibrium state. The CDP proposes that biological systems exist in constrained non-equilibrium states where variability patterns are coupled across spatial scales through the constraint architecture. Local perturbations alter local variability, which propagates through the constraint network, producing correlated changes in distant components. It doesn’t require the transfer of molecules or energy in the conventional sense, but rather the correlation of variability patterns through shared constraint boundaries.

In biological contexts, local disturbances can influence distant processes through cascading effects that exploit inherent fluctuations (Ilan 2023a, b, c, d). It fundamentally challenges traditional mechanistic views by illustrating how constraints on randomness produce highly ordered, information-rich states enabling rapid signal transmission across organizational levels.

At the molecular level, CDP explains allosteric networks in which local binding events induce distant modifications (Ilan 2023a, b, c, d; Rauscher and Pomès 2012; Wankowicz and Fraser 2025). In membrane dynamics, local compositional changes alter fluidity patterns that spread laterally across large domains (Casares et al. 2019). The mitochondrial application demonstrates how controlled variability contributes to system robustness(Ilan 2022a, b, c, d; The 2024).

In ecological systems, CDP explains how local environmental changes trigger ecosystem-wide responses through food-web dynamics (Harvey et al. 2017). CDP proposes that biodiversity represents constrained disorder, in which species interactions form constraint networks. Local extinction events alter these constraints, producing propagating effects throughout the community. This explanation predicts: (1) ecosystems with more diverse constraint networks should show greater resilience, (2) perturbations affecting keystone species (significant constraints) should produce larger-scale effects, and (3) response patterns should reflect constraint architecture rather than simple spatial proximity.

### Consciousness and the CDP

CDP is also relevant to neural network phenomena, where constrained disorder in synaptic connectivity enables information-processing capabilities that exceed those of deterministic wiring(Schug et al. 2021). Neural criticality—between order and chaos—exemplifies how biological systems utilize constrained disorder to optimize information transmission (Heiney et al. 2021). These patterns are functionally coupled to cognitive functions, memory formation, and behavioral coordination (Gelman et al. 2022)—meaning that disrupting them disrupts these functions, suggesting they are mechanistically necessary components.

Recent work explores CDP applications to consciousness itself. According to CDP, the brain exhibits a disorder with dynamic borders(Sigawi et al. 2024a, b, c). It suggests consciousness might emerge from controlled variability in neural networks. Notably, the CDP perspective on consciousness focuses on how conscious systems maintain adaptive variability within constraints, **not** on whether consciousness can influence external physical systems. It provides a more parsimonious explanation for complex biological phenomena without invoking nonlocal or non-physical mechanisms.

### Therapeutic Applications and Digital Medicine

The CDP has implications for therapeutic interventions and digital medicine. Disease states may arise from insufficient or out-of-range variability(Ilan 2019a, b, c, d, e, 2021a, b, 2023a, b, c, d, 2024a, b, c, d; The 2024; Sigawi et al. 2024a, b, c; Kessler et al. 2020; Ishay et al. 2021a, b; Kolben et al. 2021, 2023; Kenig et al. 2021; Azmanov et al. 2021, 2022; Potruch et al. 2020; Isahy and Ilan 2021; Khoury and Ilan 2019, 2021; Kenig and Ilan 2019; Gelman et al. 2020, 2022; Ilan and Spigelman 2020; Hurvitz et al. 2021, 2022, 2024; Lehmann and Ilan 2023; Adar et al. 2023). Regulating variability levels can improve system functionality (Ilan 2020a, b, c, d, 2021a, b, 2022a, b, c, d; Bayatra et al. 2024; Hurvitz and Ilan 2023; Sigawi and Ilan 2023; Sigawi et al. 2023; Landau et al. 2025). Applications include respiratory therapy, anti-aging interventions, and personalized medicine algorithms(Ilan 2019a, b, c, d, e, 2023a, b, c, d; Hurvitz et al. 2024, 2025; Sigawi et al. 2023, 2024a, b, c; Gelman et al. 2023). CDP-based management algorithms may enhance therapeutic effectiveness by working with natural biological variability.

**Fig. 2:**
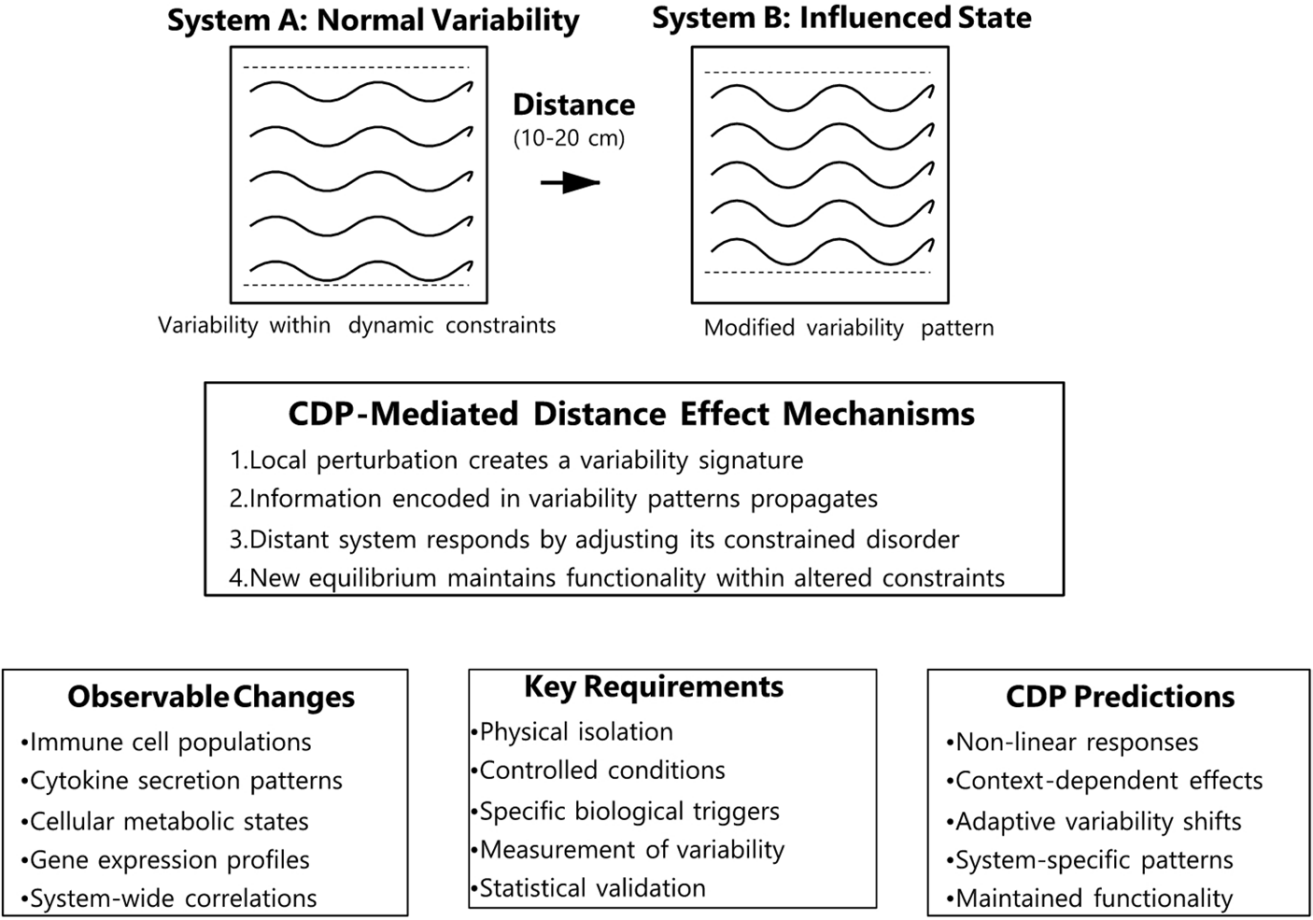
This diagram illustrates how variability, guided by the Constrained Disorder Principle, can effectively elucidate biological effects over extensive distances

Figure 2 illustrates how variability rooted in CDP can effectively elucidate biological effects observed over extensive distances. By analyzing patterns of variability captured in CDP, we gain insights into how these fluctuations influence ecosystems, thereby allowing us to understand the interconnectedness of biological responses across vast areas.

### Future Directions and Research Standards

#### Interpretation of Variability Observations

Variability patterns consistent with CDP should be interpreted carefully. While CDP provides a coherent framework for understanding these patterns, variability observations do not directly demonstrate the mechanisms underlying long-distance effects. Instead, CDP-informed variability can be interpreted as a potentially significant contributor to how biological processes manifest over distance. The framework suggests a nuanced interplay between localization and broader system contexts, but establishing causal mechanisms requires additional targeted experimentation.

#### Testable Predictions and Future Experiments

To move from framework to verified mechanism, future CDP research should test specific predictions.

1. **Constraint manipulation**: If CDP explains distance effects through constraint networks, experimentally altering constraints (not just components) should predictably modify distance correlations.
2. **Variability pattern transfer**: If variability patterns carry information, matching variability signatures between systems should enhance correlations even without molecular mediators.
3. **Scale invariance**: CDP predicts that similar principles operate across scales—distance effects should show comparable patterns from cellular to organismal to ecosystem levels.
4. **Temporal dynamics**: Constraint propagation should show characteristic time courses distinct from diffusion or neural conduction.

These predictions provide specific experimental approaches to test whether CDP genuinely explains distance effects or merely describes them.

Future research on controversial distance effects should adhere to the highest methodological standards: pre-registration, adequate statistical power, appropriate controls, blinding protocols, and plans for independent replication. New technologies for detecting subtle biological effects offer opportunities for more definitive testing of controversial hypotheses. However, technological advances must be coupled with rigorous methodology to distinguish genuine phenomena from artifacts. The goal should be to maintain scientific openness while upholding the standards necessary to advance reliable knowledge(Ilan 2024a, b, c, d, 2025a, b, c, d, e; Landau et al. 2025).

## Replication, Methodological Rigor, and the Debate on Long-Distance Effects

Evaluating extraordinary claims about biological distance effects requires adherence to rigorous scientific standards(Tressoldi 2011): appropriate experimental controls, blinding protocols, statistical power analysis, hypothesis pre-registration, and independent replication by multiple research groups. The challenge lies in distinguishing genuine phenomena from experimental artifacts, statistical fluctuations, and investigator bias(Ilan 2024a, b, c, d, 2025a, b, c, d, e; Landau et al. 2025).

### Scientific Demarcation Criteria Applied

The broader scientific community recognizes replication challenges across research fields. Studies of marginal effects are particularly vulnerable to methodological issues. For extraordinary biological phenomena, including consciousness effects and nonlocal correlations, multiple criteria must be satisfied.

1. **Reproducibility**: Independent laboratories using identical protocols should obtain similar results.
2. **Effect size**: Effects must exceed measurement error and show a consistent magnitude.
3. **Statistical rigor**: Appropriate power analysis, pre-registration, correction for multiple comparisons, and protection against p-hacking.
4. **Mechanistic plausibility**: Proposed mechanisms should be consistent with established physical principles or provide compelling reasons to revise them.
5. **Alternative explanations**: Simpler explanations (artifacts, bias, chance) must be ruled out.
6. **Theory integration**: Findings should connect to broader theoretical frameworks.

The phenomenon of p-hacking, in which analyses are adjusted to achieve statistical significance, can lead to spurious findings that fail to replicate. It is particularly pronounced in studies of marginal effects where small biases shift results across significance thresholds.

Studies exploring the effects of consciousness and other extraordinary biological phenomena are especially vulnerable to these methodological challenges. The combination of small effect sizes, high investigator motivation, and limited replication attempts creates an environment in which false-positive findings can persist (Vankelecom et al. 2025). Transparent reporting of borderline conclusions is essential for scientific integrity, but such results require substantially more evidence before firm conclusions can be drawn (Weiss et al. 2023). The scientific community appropriately maintains skepticism toward unreplicated marginal effects while remaining open to compelling new evidence that meets rigorous standards.

## Conclusion

This review of distance effects in biology highlights a clear distinction between well-established phenomena and extraordinary claims. **Historical perspective**: The history of biology includes numerous examples of initially poorly explained distance effects that were accepted or rejected on the basis of rigorous criteria. For example: (1) **Accepted**: Hormonal signaling was initially mysterious “chemical messengers” but became understood through isolation of specific molecules, identification of receptors, and elucidation of signaling pathways meeting all demarcation criteria. (2) **Rejected**: “Odic force” (mid-1800s) proposed invisible emanations from magnets and living things affecting distant objects—failed replication and lack of physical mechanism led to rejection. (3) **Accepted**: Electromagnetic field effects on development were initially controversial but became accepted through reproducible experimental protocols, identification of specific ion channels and voltage gradients, and a consistent mechanistic framework (bioelectricity). These examples illustrate how legitimate distance effects transition from speculation to acceptance by satisfying the demarcation criteria outlined in this review. At the same time, unsubstantiated claims fail to make this transition despite persistent advocacy.

Conventional biological distance effects, mediated by known physical mechanisms such as diffusion, electromagnetic fields, and cellular transport, have robust experimental support and a solid theoretical foundation. Claims regarding consciousness-mediated distance effects, morphic resonance, and similar phenomena lack convincing experimental evidence and theoretical justification. Emoto’s work on water crystallization, though popular, does not meet basic scientific standards and has not been successfully replicated under controlled conditions. Similarly, proposals for quantum field effects or morphic fields as mediators of biological distance communication remain speculative and lack empirical support.

The CDP offers a promising framework for understanding biological complexity and its effects on distance. By focusing on controlled variability in biological systems, CDP provides insights into adaptation and therapeutic intervention without invoking non-physical mechanisms. The novel experimental findings on nonlocal immune correlations presented in Sect. 5.4 motivate the CDP framework and suggest new directions for understanding biological organization. However, substantial additional research meeting rigorous replication standards is needed before these findings can be considered definitively established.

The debate over extraordinary claims in biology emphasizes the importance of methodological rigor and replication in scientific research. While remaining open to new possibilities, the scientific community must demand extraordinary evidence for extraordinary claims. Future research should concentrate on deepening the understanding of established distance effects while rigorously testing extraordinary claims. The objective should be to expand scientific knowledge while maintaining epistemological standards that distinguish science from speculation. By adhering to these principles, research on biological distance effects can continue providing valuable insights into the fundamental nature of living systems.

## Data Availability

No datasets were generated or analysed during the current study.

## Abbreviations

CDP: constrained disorder principle
AI: artificial intelligence
